# Inhibition of cyclooxygenase-2 by NS398 attenuates noise-induced hearing loss in mice

**DOI:** 10.1038/srep22573

**Published:** 2016-03-03

**Authors:** Yu Sun, Jintao Yu, Xi Lin, Wenxue Tang

**Affiliations:** 1 Department of Otolaryngology, Union Hospital, Tongji Medical College, Huazhong University of Science and Technology, Wuhan 430022, PR China; 2 Department of Otolaryngology, Emory University School of Medicine, 615 Michael Street, Atlanta, GA 30322-3030, USA; 3 Department of Otolaryngology, The Second Affiliated Hospital of Zhengzhou University, Zhengzhou 450014, PR China

## Abstract

Noise-induced hearing loss (NIHL) is an important occupational disorder. However, the molecular mechanisms underlying NIHL have not been fully clarified; therefore, the condition lacks effective therapeutic methods. Cyclooxygenase-2 (Cox-2) is an inducible enzyme involved in the synthesis of prostaglandins, and has been implicated in many pathophysiological events, such as oxidative stress and inflammation. In this study, we investigated the possible role of Cox-2 in the mechanisms of NIHL and the therapeutic effect of the Cox-2 inhibitor NS398 on NIHL using a mouse model. We demonstrated that Cox-2 is constitutively expressed in the mouse cochlea, and its expression could be dramatically up-regulated by high levels of noise exposure. Furthermore, we demonstrated that pre-treatment with the Cox-2 inhibitor NS398 could inhibit Cox-2 expression during noise overstimulation; and could attenuate noise-induced hearing loss and hair cell damage. Our results suggest that Cox-2 is involved in the pathogenesis of NIHL; and pharmacological inhibition of Cox-2 has considerable therapeutic potential in NIHL.

Noise-induced hearing loss (NIHL) is one of the most prevalent occupational disorders in the world. It is estimated that approximately 16% of cases of adult-onset hearing loss are caused by NIHL worldwide^1^. This percentage is even higher in developing countries^2^,^3^. NIHL has a great effect on workers’ speech communication and leads to accidents and injuries in the workplace^4^. The high prevalence of NIHL is a huge burden to society. Therefore, attenuating or preventing NIHL are of interest to researchers, and there has been great effort to understand the molecular and biochemical mechanisms underlying NIHL.

Studies have demonstrated that loud noise overstimulation could cause a broad set of physiologic and biochemical changes in the cochlea leading to the cell death. High levels of noise could lead to the excessive release of the excitatory neurotransmitter glutamate at the synapse between the inner hair cells (IHCs) and spiral ganglion neurons (SGNs), causing glutamate excitotoxicity^5^. Excess glutamate in the extracellular space and activation of the glutamate receptors induces an overload of intracellular Ca^2+^ levels, which in turn triggers a cascade of metabolic events, including enhanced free-radical production, protease and endonuclease activation, and mitochondrial dysfunction, ultimately leading to cell death^6^,^7^,^8^,^9^. Additionally, overexposure to high intense noise could lead to excess production of reactive oxygen species (ROS) in the cochlea, causing oxidative stress, which has been demonstrated to play a fundamental role in NIHL^10^,^11^. High levels of ROS could be observed immediately and several days after noise exposure in the cells of the cochlea, and these high levels of ROS are accompanied by depletion in cellular antioxidant enzyme levels^12^,^13^,^14^. The increased ROS could react with biological macromolecules producing lipid peroxidation, DNA damage and enzyme inactivation, triggering multiple cell death pathways^15^. High levels of noise exposure could also induce inflammatory responses in the cochlea^16^,^17^,^18^. The recruitment of circulating leukocytes and up-regulation of pro-inflammatory cytokines such as TNF-α, IL-1β and IL-6 in the cochlea were observed following noise exposure^17^,^19^. Pathological processes mediated by inflammatory cells and cytokines also contribute to cochlear cell death after acoustic injury^20^. Despite these findings, the mechanisms underlying NIHL have not been fully clarified.

Cyclooxygenases (COXs), also named prostaglandin endoperoxide synthases, are the key enzymes involved in the first step of the synthesis of a series of prostaglandins and leukotrienes from arachidonic acid^21^. There are at least two isoforms of Cox, Cox-1 and Cox-2, which have quite different expression patterns and biological functions. Cox-1 is a constitutively expressed protein found in most tissues, whereas Cox-2 is a inducible enzyme that could be up-regulated by various cytokines, growth factors and endotoxins in numerous tissues^21^. Cox-2 is considered to be a crucial mediator of many pathological conditions. Studies have demonstrated that Cox-2 is involved in mechanisms of neuronal cell death induced by glutamate excitotoxicity, cerebral ischemia, and neuroinflammation in the brain. Up-regulation of Cox-2 expression and enzymatic activity promotes neuronal injury in a number of acute and chronic brain disorders such as stroke, traumatic brain injury, Parkinson’s disease, and amyotrophic lateral sclerosis^22^,^23^,^24^, and inhibition of Cox-2 activity has been shown to be neuroprotective^25^. Therefore, Cox-2 inhibitors have been proposed to have potential therapeutic applications for treating these disorders. Recently, the distribution of Cox-2 in guinea pig and human cochlea has been described^26^,^27^. However, the role of Cox-2 in hearing and possibly pathological processes in the inner ear remains unknown. One study showed that Cox-2 expression in the organ of Corti could be down-regulated by moderate sound exposure designed to produce sound conditioning, and the author hypothesized that the alteration of Cox-2 might be part of the sound conditioning effect (sound conditioning refers to the acquired resistance to NIHL caused by pre-exposure to low level non-traumatic noise)^10^,^28^. This finding suggests a potential role for Cox-2 to mediate the responses of cochlea to acoustic injury. Therefore, Cox-2 activation might be involved in the pathogenesis of NIHL. To test this hypothesis, in the current study, we explored the possible role of Cox-2 in NIHL. We investigated whether NIHL is associated with up-regulation of the Cox-2 and if so, we sought to define whether Cox-2 expression contributes to noise-induced hearing threshold shifts and hair cell death by the pharmacological inhibition of Cox-2 expression.

## Results

### Cox-2 is expressed in the mouse cochlea

**Figure 1:**
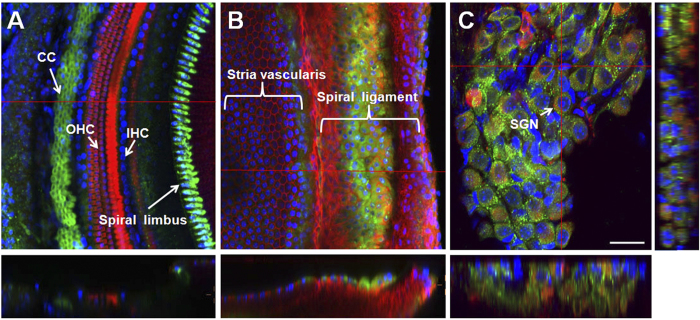
The expression and localization of Cox-2 in the mouse cochlea. Cox-2 was immunolabeled with primary antibody against Cox-2 and Alexa Fluor 488-conjugated secondary antibody (green). The hair cells as well as the marginal cells (MC) and the fibrocytes (FC) of the cochlear lateral wall were labeled with Alexa Fluor 568-conjugated phalloidin (red). Spiral ganglion neurons (SGN) were labeled with primary antibody against neurofilament and TRITC-conjugated secondary antibody (red). The nuclei were labeled with DAPI (blue). (**A**) Cox-2 expression in the cells of the organ of Corti. (**B**) Cox-2 expression in the cochlear lateral wall. (**C**) Cox-2 expression in the cochlear SGNs. Scale bar shown in the figure represents 25 μm.

To explore the expression and localization of Cox-2 in the mouse cochlea, immunostained cochlear whole mounts were studied by confocal microscopy. Our data demonstrated that Cox-2 is constitutively expressed in the mouse cochlea (Fig. 1). However, the immunostaining pattern of Cox-2 varies in different regions of the cochlea. In the organ of Corti, strong Cox-2 staining reaction was observed mainly in the supporting cells. As shown in Fig. 1A, marked immunostaining was noted in the Claudius cells (CCs), and week staining was observed in the Hensen’s cells (HCs), Deiter’s cells (DCs) and Border cells (BCs). Negative or only a faint staining was demonstrated in the sensory hair cells, including IHCs and outer hair cells (OHCs), and Pillar cells (PCs). The expression pattern of Cox-2 in the organ of Corti along the entire cochlear spiral was homogeneous. In the region of the cochlear lateral wall, an intense staining reaction was observed in the spiral ligament, and weaker staining was demonstrated in the stria vascularis (Fig. 1B). A distinct Cox-2 expression pattern in spiral ligament was identified, and this pattern showed a strong reaction in cochlear middle turn, a weaker reaction in the apical turn and a lower reaction in the basal turn. In the region of the cochlear spiral limbus and spiral ganglion neurons, an intense Cox-2 immunoreaction was also observed (Fig. 1A,C). However, a gradual decrease of Cox-2 expression from the cochlear basal turn to the apical turn was found in both regions.

### Cox-2 protein expression is up-regulated during noise exposure

**Figure 2:**
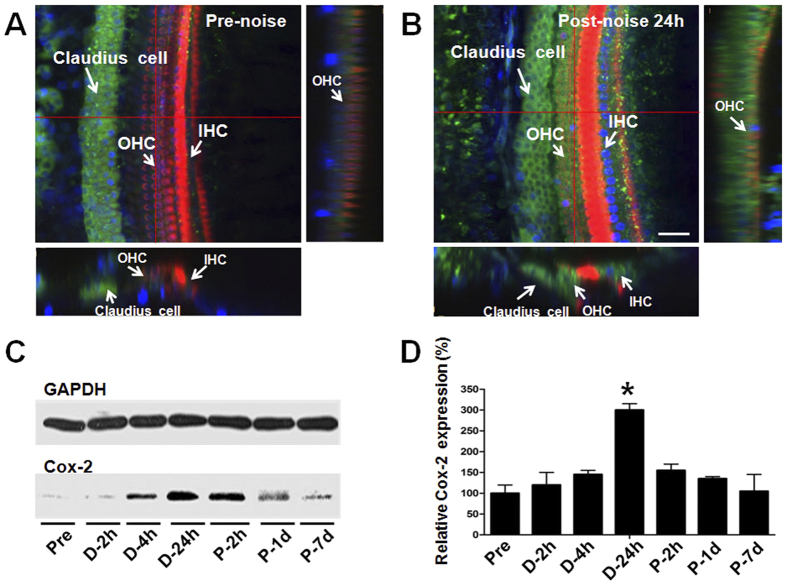
Cox-2 protein expression changes induced by noise exposure. (**A**,**B**) Representative confocal photomicrographs showing the Cox-2 expression in the organ of Corti before and 24 h after noise exposure. Cox-2 was immunolabeled with primary antibody against Cox-2 and Alexa Fluor488-conjugated secondary antibody (green). The cochlear hair cells were labeled with Alexa Fluor 568-conjugated phalloidin (red). The nuclei were labeled with DAPI (blue). Scale bar represents 25 μm. (**C**,**D**) Western bolts showing the expression levels of Cox-2 in the mouse cochlea at various time points before, during, and after noise exposure. The Cox-2 protein level was significantly increased at 24 h during noise exposure (D-24h) and gradually recovered 7 days post-noise exposure (P-7d). *P < 0.01 compared with the Pre level.

To investigate the response of Cox-2 expression in the mouse cochlea to acoustic injury, the Cox-2 protein level was evaluated by immunofluorescent staining and western bolt analysis at various time points before, (2, 4, 24 h) during and (4 h, 1, 7 days) after noise exposure. We found that the expression level of Cox-2 was significantly up-regulated by noise stimuli. In the organ of Corti especially in the cochlear hair cells significant Cox-2 expression was observed after noise exposure (Fig. 2A,B). However, this up-regulation gradually disappeared several days post noise treatment. As shown in Fig. 2C,D, an increased Cox-2 protein level was detected rapidly at 2 h during noise exposure, and by 24 h the highest protein level was observed (P < 0.01). Then, after noise exposure a down-regulation of the Cox-2 protein level was seen; by 7 days post-exposure, the Cox-2 protein expression mostly recovered to the basal level. These results suggested a time-dependent change in the expression of Cox-2 in response to noise overexposure.

### NS398 inhibits Cox-2 protein expression induced by noise exposure

**Figure 3:**
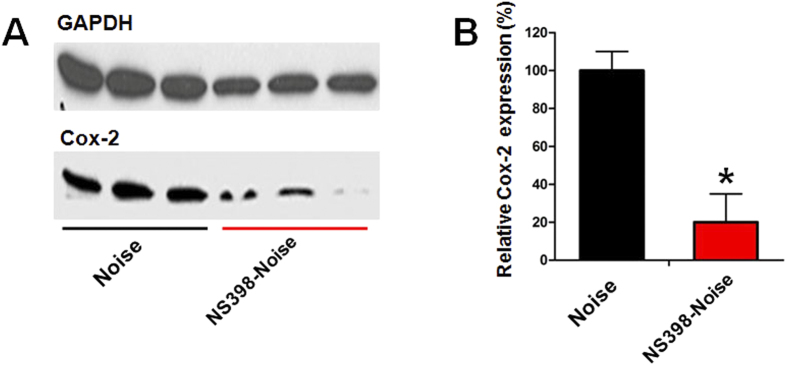
NS398 inhibits Cox-2 protein expression induced by noise exposure. (**A**) Representative western bolts showing the expression levels of Cox-2 in the noise and NS398-Noise groups following 24 h of noise treatment. (**B**) The Cox-2 protein level was significantly decreased in the NS398-Noise group after noise exposure. *P < 0.01 compared with the noise group.

To determine the effect of the Cox-2 inhibitor NS398 on the expression of Cox-2 during noise exposure, animals were pre-treated with NS398 before noise exposure and Cox-2 protein level was examined by immunoblotting at 24 h during noise treatment (see protocol 3 in materials and methods). This time point was selected because it was the point at which the Cox-2 protein elevation was the highest. Figure 3A shows the western blot analysis data of Cox-2 expression in the saline-noise and NS398-noise groups. Pre-treatment with NS398 resulted in a great reduction of the Cox-2 protein band intensity compared with the saline-noise control. The quantitative analysis revealed that the protein level of Cox-2 in the NS398-noise group was only 27% of the saline-noise control after noise exposure (Fig. 3B). The reduction was statistically significant (P < 0.01). These results demonstrated that pre-treatment with NS398 inhibited Cox-2 expression induced by noise overstimulation.

### NS398 attenuates the noise-induced hearing threshold shift

**Figure 4:**
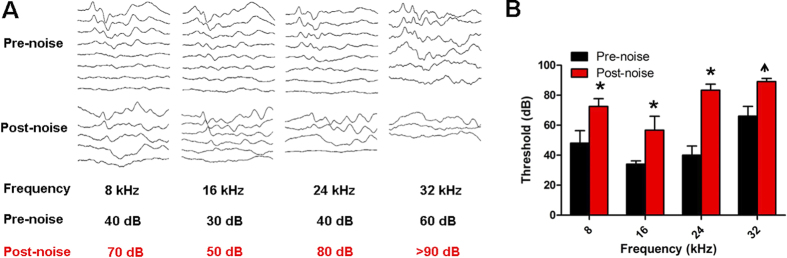
Hearing thresholds and representative ABR waveforms at different frequencies before and 7 days after noise exposure. In the (panel **B**), vertical bars represent SD. n = 6 per group. *P < 0.05 compared with the pre-noise level. Up arrow on post-noise data indicate that threshold is underestimated, because no response was detected at the highest level presented at 90 dB SPL by ABR test.

**Figure 5:**
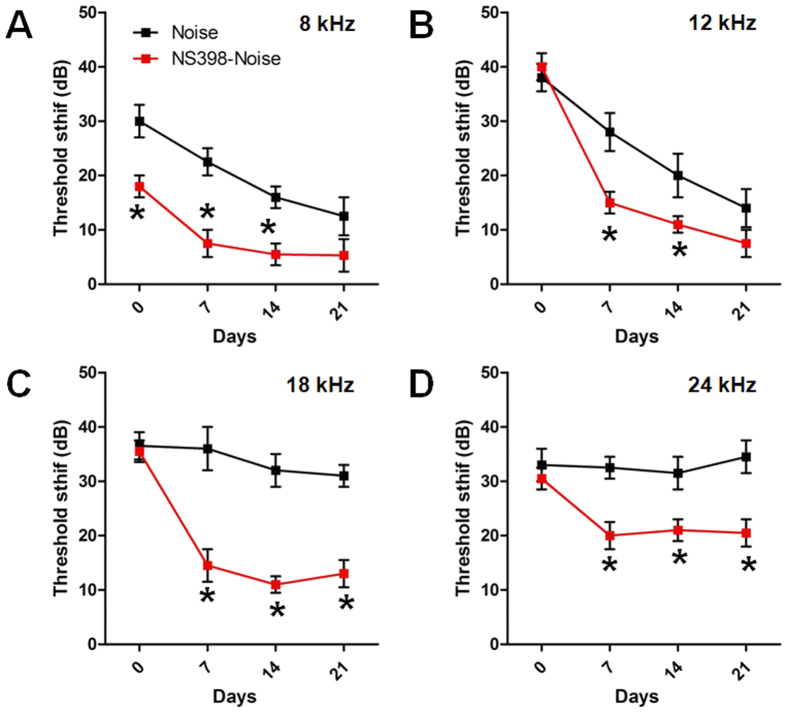
The ABR threshold shifts in the noise and NS398-Noise groups after noise exposure at different frequencies. *P < 0.05 compared with the noise group.

To evaluate the effect of Cox-2 inhibition on NIHL, the hearing thresholds of mice in the control group, noise group and NS398-noise group were determined at different time points by ABR measurement (see protocol 4 in materials and methods). The mean initial thresholds of the experimental animals at the frequency of 8 kHz, 16 kHz, 24 kHz, and 32 kHz were 48 ± 8.367 dB, 34 ± 2.236 dB, 40 ± 6.124 dB, and 66 ± 6.519 dB, respectively, which shows hearing impairment at the high frequency. The results were similar with previous studies^29^,^30^,^31^. After noise exposure, great threshold elevation was observed across all test frequencies; the ABR signal became undetectable at 32 kHz 7 days after noise exposure (Fig. 4). In order to avoid the influence of this early high frequency hearing impairment on the results of the NS398’s protective effect, only low and middle hearing frequencies (8, 12, 16 and 24 kHz) were chose for functional evaluation. As shown in Fig. 5, immediately after noise exposure, mice in the noise group developed approximately 30–40 dB threshold shifts across all test frequencies. In the NS398-noise group, similar threshold shifts were observed at high frequencies of 12, 18 and 24 kHz; however, the threshold shift at 8 kHz was significantly lower than that of the noise group (P < 0.05). With the recovery of hearing sensitivity after noise exposure, the difference between the two groups became evident. The threshold shifts in the NS398-noise group recovered rapidly and showed a significant reduction in threshold shifts especially at high frequencies of 18 and 24 kHz compared with the noise group measured at 1, 2, and 3 weeks after noise exposure (P < 0.01). At the most pronounced frequency of 18 kHz, the NS398-noise group exhibited up to a 20 dB lower threshold shift than the noise group. This result indicated that NS398 pre-treatment could reduce NIHL.

### NS398 protects against noise-induced hair cell loss

**Figure 6:**
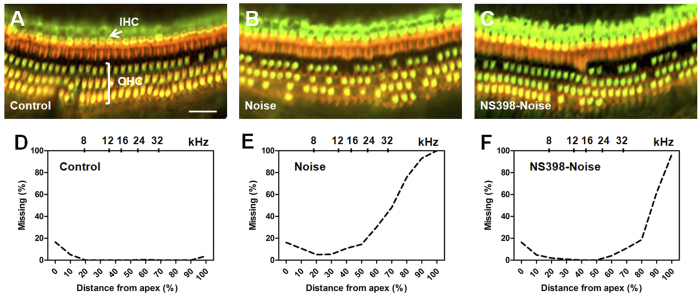
Cochlear hair cell loss in different groups 21 days after noise exposure. (**A**–**C**) Examples of cochlear hair cells at a location related to 24 kHz (approximately 52% from the apex) in the different groups. Hair cell bodies were labeled with primary antibody against myosin-VIIa and Alexa Fluor488-conjugated secondary antibody (green). Hair cell stereocilia and cuticular plate were labeled with Alexa Fluor 568-conjugated phalloidin (red). Scale bar represents 20 μm. (**D–F**) Mean cochleograms (n = 5) for each treatment group show the degree of hair cell loss as a function of the percent distance from the apex of the cochlea.

**Figure 7:**
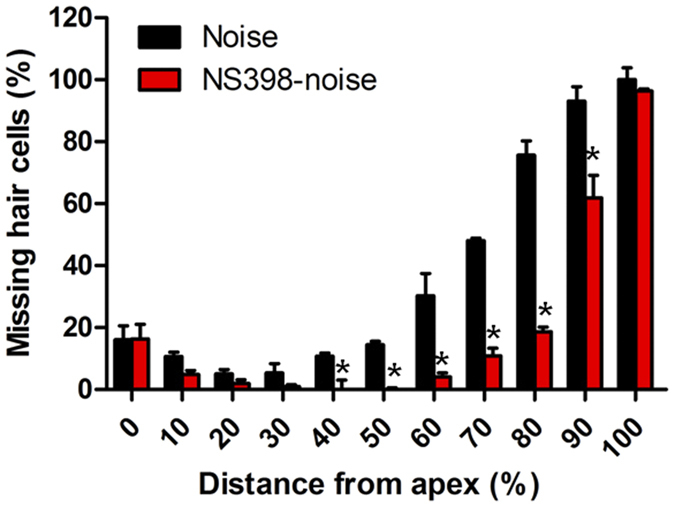
Comparison of the hair cells loss between the noise and NS398-Noise groups 21 days after noise exposure. *P < 0.05 compared with the noise group.

To further determine if Cox-2 inhibition had a protective effect on hair cell survival, cochlear hair cell loss was evaluated after noise exposure (see protocol 4 in materials and methods). No hair cell loss or mild hair cell loss was observed along the entire cochlear spiral in the control group despite hearing impairment was revealed at the high frequency of 32 kHz. The results were consistent with previous reports^29^,^31^ (Fig. 6A,D). However, obvious hair cell loss was found in the noise and NS398-noise groups by 3 weeks after noise exposure (Fig. 6B,C). Both of the two noise exposed groups showed a similar pattern of hair cell loss, which was found mainly focused in the basal region of the cochlea and the OHC loss was more severe than the IHC loss. This histological finding also corresponds with the physiological data in which the hearing threshold shift was more severe at high frequencies. The mean cochleograms (n = 5) for the different groups were shown in Fig. 6D–F; percent distance from the apex is related to frequency using a mouse frequency-place map^32^. Obvious hair cell loss in the noise group occurred starting from a 20% distance from the apex of the cochlea (near the 8 kHz region), whereas in the NS398-noise group, evident hair cell loss was not found until a 50% distance from the apex (near the 24 kHz region). The quantitative analysis revealed that hair cell loss in the NS398-noise group was significantly less than that of the noise group (Fig. 7). This result suggested that NS398 pre-treatment provides significant protection against noise-induced hair cell loss.

## Discussion

In the present study, we used a mouse model to investigate the role of Cox-2 in the mechanisms of NIHL. We found that Cox-2 was constitutively expressed in the mouse cochlea, and its expression could be dramatically up-regulated by high levels of noise exposure. We demonstrated that pre-treatment with the Cox-2 inhibitor NS398 could inhibit Cox-2 expression induced by acoustic injury and could attenuate noise-induced hearing threshold shifts and cochlear hair cell loss. These findings provide strong evidence that Cox-2 activation is involved in the pathogenesis of noise-induced damage in the cochlea. Our findings also indicate that pharmacological inhibition of Cox-2 has considerable therapeutic potential in NIHL.

The CD-1 mouse strain is known exhibiting an early onset of hearing loss that appears first at high frequencies and progressively involved lower frequencies, which makes it as an interesting model to investigate the mechanisms of age related hearing loss^29^,^33^,^34^. In the present study, we choose CD-1 mouse as the experimental subject is mainly based upon the consideration that it might be more sensitive to NIHL as prior studies have shown that some strains of mice showing hearing impairment early in life appear to be more susceptible to NIHL^35^,^36^. While the results of noise-induced cochlear damage in the current experiment could be influenced by the early hearing loss occurred at the high frequency, we believe this is unlikely since there are studies have shown that the cochlear surface preparation often remained normal until about 3–4 months of age, despite hearing loss occurred early in CD-1 mice^29^,^31^. Consistently, our results also demonstrated intact cochlear hair cells along the entire cochlear basilar membrane in the control group (Fig. 6). In addition, the hearing thresholds at low frequencies present normal in the early life of CD-1 mice^31^. In our study, because of the undetectable ABR signals at 32 kHz after noise exposure due to the age-related hearing loss, low frequencies were tested to evaluate the functional changes. Thus, with the well prepared control, we believe the early high frequency hearing impairment has minmal influence on the results of hair cell counts and functionally evaluation especially at the low frequencies. However, it would be helpful to use an inbred mouse strain with normal hearing, such as CBA mouse, to further evaluate the effects at the high frequencies.

The immunostaining data in the current study showed that Cox-2 is constitutively expressed in most cell types in the mouse cochlea, with much stronger expression in the supporting cells of the organ of Corti and SGNs. Our results are similar to the findings reported in the guinea pig cochlea^27^,^28^, indicating that Cox-2 might have important physiologic functions related to hearing. However, the function of Cox-2 in the cochlea remains unknown. Studies in other tissues and related diseases have demonstrated that Cox-2 is primarily involved in producing prostaglandins in response to a wide spectrum of environmental insults and internal stimuli^21^. In the current study, we found a time-dependent change of Cox-2 protein expression in the cochlea following noise overstimulation. The results demonstrated that the Cox-2 protein level was significantly increased during noise exposure, beginning at 2 h and peaking at 24 h, and then gradually returned to the baseline after noise exposure (Fig. 2). These data provide evidence that Cox-2 levels in the mouse cochlea could be dramatically induced by intense noise overexposure. The most recent study also showed Cox-2 in the organ of Corti could be up-regulated by gentamicin treatment^37^. Collectively, these findings suggest that Cox-2 in the cochlea might serve as an important modulator of cochlear responses to various ototoxic insults, such as loud noises and ototoxic drugs.

In the current study, we demonstrated that pre-treatment with the Cox-2 inhibitor NS398 reduced Cox-2 expression level during noise exposure and attenuated noise-induced hearing loss and hair cell damage. These results indicated that Cox-2 up-regulation is involved in the pathogenesis of NIHL, although the exact role of Cox-2 in NIHL remains unknown. ROS-mediated oxidative stress has been regarded as an important mechanism of noise-induced cell death in the cochlea^10^,^11^,^38^. Acoustic overstimulation could increase intra-cochlear ROS, which could react with cellular constituents, such as lipids, proteins and nucleic acids, triggering multiple signal pathways leading to cell death^12^,^39^. A variety of antioxidants have been demonstrated to effectively reduce sensory cell death and NIHL in animal models^38^,^40^,^41^. Cox-2 expression has been demonstrated to have a direct association with the formation of ROS^42^. ROS could be produced by Cox-2 reactions that catalyze the conversion of arachidonic acid to prostaglandin H2^43^. Cox-2 derived ROS have been implicated in the cellular damage involved in many pathological conditions in the brain^43^,^44^. Therefore, noise-induced up-regulation of Cox-2 in the inner ear might also contribute to the damage of cochlear cells caused by oxidative stress. This hypothesis was supported by a recent study showing that the antioxidant resveratrol could reduce NIHL by decreasing Cox-2 expression and related ROS formation^45^.

In the central nervous system, Cox-2 has been demonstrated to play a crucial role in glutamate mediated excitotoxicity. Overexpression of neuronal Cox-2 could potentiate the intensity and lethality of glutamate excitotoxicity^44^,^46^, and inhibition of Cox-2 protects neurons from glutamate-mediated cell death^47^. Because intense noise exposure could also induce excessive release of glutamate in the cochlea, these findings indicate that Cox-2 might participate in noise-induced glutamate excitotoxicity in the inner ear. This hypothesis could be supported by our immunostaining data that Cox-2 is strongly expressed in auditory neurons (Fig. 1C), and the expression might be modulated by exitotoxic concentrations of glutamate, as have been described in the brain^48^. Cox-2 is generally accepted to be an important mediator of inflammation in numerous tissues including the inner ear^49^, and high levels of noise overstimulation could induce immune cell invasion of the cochlea and increase pro-inflammatory cytokines causing inflammatory responses^19^,^50^. In the study by Hirose *et al.*, after noise overexposure, CD45^+^ mononuclear phagocytes were found to be mainly concentrated in cochlear spiral ligament and spiral limbus, which was where Cox-2 was strongly expressed in the current experiment, indicating that Cox-2 might play a role in propagating cellular damage caused by cochlear inflammation^50^. Based upon this information, we hypothesize that Cox-2 might serve as an important mediator of multiple pathophysiologies in the cochlea triggered by noise overstimulation, and up-regulation of Cox-2 might participate in the mechanisms of cellular damage in NIHL. Cox-2 inhibition likely attenuates NIHL and hair cell damage by reducing noise-induced oxidative stress, glutamate excitotoxicity and inflammatory responses.

In summary, we demonstrated that Cox-2 is involved in the pathogenesis of NIHL, and inhibition of Cox-2 by NS398 could attenuate NIHL and related hair cell damage. However, the exact role of Cox-2 in NIHL remains unknown, further studies are warranted to clarify this issue.

## Methods

### Subjects and overall design

Eighty-four healthy female CD1 mice aged 1–2 months were used for this study (Charles River Laboratories). The experiments were performed according to the National Institutes of Health Guidelines for Experimental Animals and were approved by the Animal Care and Use Committee at Emory University. Four separate experiments were performed according the following protocols. (1) Three animals were used to study the immunohistochemical locations of Cox-2 in adult mouse cochlea. (2) Forty-eight animals were used to determine cochlear Cox-2 expression responses to noise exposure. Cochleae were collected, and the Cox-2 protein level was examined at several time points (pre-, 2, 4, 24 h during and 4 h, 1, 7 days post-noise exposure) by western bolt and immunostaining (six animals at each time point). (3) Eight animals were used to assess the effect of the Cox-2 inhibitor-NS398 on the expression of Cox-2 during noise exposure. The animals were randomly divided into two groups (four animals in each group): a NS398-noise group, in which mice were pretreated with 20 mg/kg NS398 diluted in normal saline twice over a 1 h interval by intraperitoneal injections; and a noise group, in which the mice were given the same volume of vehicle (normal saline) over the same schedule. After being exposed to 115 dB SPL continuous broadband noise for 24 h, mice in the two groups were sacrificed, and both sides of the cochlea in each mouse were used for Cox-2 immunoblotting. (4) Twenty-four animals were used to evaluate the effect of Cox-2 inhibition on NIHL. The animals were divided among three groups: a control group, noise group and NS398-noise group. Mice in the NS398-noise group or mice in the control and noise groups were pretreated with NS398 or saline as describe above. Then, mice in the noise and NS398-noise groups were exposed to 115 dB SPL continuous broadband noise for 24 h. Mice in the control group were sham exposed with the same schedule. After noise exposure, the animals’ hearing thresholds were examined by auditory brainstem response (ABR) immediately and 1, 2, 3 weeks post-noise exposure, and cochlear hair cell loss was examined.

### Noise exposure

Noise exposure was performed in a sound booth with animals housed in separate wire cages (30 × 30 × 15 cm) located 20 cm in front of two loudspeakers (Eminence, USA). The acoustic stimulus was generated digitally using a real-time signal processor (Tucker Davis Technologies, TDT, USA), and routed through an attenuator (TDT, USA) and a power amplifier (Crown XLS 202, Harman International Company, USA) to the loudspeakers. The overall noise levels within each cage were measured at multiple locations with a sound level meter (Larson-Davis, LD 800B) placed at a height equal to the level of the animals’ head. The exposure level varied by less than 2 dB SPL between cages. During exposure, the animals were allowed free access to food and water. After noise exposure, the animals were returned to their home cages in the animal colony.

### Cox-2 immunostaining

The animals were anesthetized with a mixture of ketamine (100 mg/kg, i.m.) and xylazine (15 mg/kg, i.m.) and sacrificed by cervical dislocation. Cochleae were quickly removed and fixed in 4% paraformaldehyde in PBS at 4 °C for 2 h. Then, the basilar membrane, the cochlear lateral wall, and the spiral ganglion neurons were microdissected out. The specimens were immersed overnight at 4 °C in a polyclonal primary rabbit anti-Cox-2 antibody (abcam, USA) diluted in 1% Triton X-100 and 5% donkey serum in 0.1 M PBS (1:100). After three washes in PBS, specimens were incubated in a secondary donkey anti-rabbit antibody conjugated with Alexa Fluor 488 diluted in PBS (1:200) for 2 h at room temperature. Then, specimens were incubated for 1 h at room temperature with Alexa Fluor 568-conjugated phalloidin (Sigma, USA) to stain the hair cell stereocilia and cuticular plate. After rinsing in PBS, the specimens were immersed for 10 min in fresh DAPI solution to label the nuclei of cells in the specimen. To mark spiral ganglion neurons, the specimens were co-incubated with a monoclonal primary mouse anti-neurofilament antibody (Sigma, USA) and a secondary donkey anti-mouse antibody conjugated with TRITC. The specimens were subsequently coverslipped with a SlowFade® anti-fading mounting solution and examined under a confocal microscope (Carl Zeiss, Germany).

### Western bolt analysis

Western blot analysis was performed as previously described^51^. The animals were anesthetized and perfused transcardially with PBS (pH 7.4). The temporal bones were quickly removed and cochlear tissues including the cochlear modiolus, auditory nerve, and sensory epithelium were carefully dissected on ice. The total protein was extracted using RIPA lysis Buffer (Upstate Biotechnology Cell Signaling System, USA), and the protein concentrations were measured using the BCA™ protein assay kit (Pierce, USA). For analysis, sample proteins (6 μg per lane) were separated by electrophoresis on a 10% sodium dodecyl sulfate (SDS) polyacrylamide gel and transferred to nitrocellulose membranes. Bands were incubated in a blocking solution (TBST containing 5% non-fat dry milk) for 1 h and then incubated with a polyclonal rabbit anti-mouse Cox-2 antibody (1:400, Cayman Chemical, USA) overnight at 4 °C. After washing with TBST, the membranes were incubated for 1 h at room temperature with a horseradish peroxidase (HRP)-conjugated goat anti-rabbit secondary antibody. The protein bands were visualized by reacting with a chemiluminescent substrate (Pierce Inc., USA), and exposing on XAR-5 films (Kodak, USA). We ensured the densitometric intensity of measured bands was in the linear range in relation to protein concentration as determined by preliminary experiments. We further verified the amount of protein loading in each lane by stripping the blots (100 mM 2-Mercaptoethanol, 2% SDS and 62.5 mM Tris pH = 6.7 at 50 °C for 30 min) and re-probing with a monoclonal antibody against GAPDH (1:4000, Chemicon international, USA). The relative density of each band was measured and analyzed by NIH Image software (version 1.61). The background in the films was subtracted from the optical density measurements.

### ABR measurement

Hearing thresholds were determined by ABR measurement. Baseline ABR thresholds for all mice were obtained first within 2 days prior to drug treatment, and then threshold changes were evaluated at several time points after noise exposure and treatment. Animals were anesthetized with a mixture of ketamine and xylazine as described above, and the body temperature was maintained at 37 °C using a heating pad. Testing was performed in a sound-isolated and electrically shielded booth. ABR responses were collected using three subcutaneous electrodes (Model E2; Grass Instruments, USA) and processed by a TDT system (Model DA3-2, Tucker-Davis Technologies, USA). Tone burst stimuli at 8, 12, 18 and 24 kHz were generated digitally with a 3 ms duration, 0.5 ms rise/fall time, alternating phase, repeat rate 21/s and fed to a speaker (Model DT-48; Beyer Dynamic, Germany) with a 10 cm plastic tube placed in the animal’s external auditory canal. The responses were filtered between 0.3 and 3.0 kHz and averaged 512 times. The thresholds were obtained by reducing the sound intensity in 5 dB increments from 90 to 5 dB SPL. For each of the test frequencies, auditory thresholds were determined by identifying the smallest intensity at which the ABR wave forms became evident.

### Hair cell examination

Animals were decapitated after functional hearing assessments. The cochleae were quickly removed from the temporal bone and perfused with 4% paraformaldehyde in PBS through the oval and round windows and were fixed in the same solution for 24 h at 4 °C. The samples were decalcified in 10% EDTA solution for 3 days. The cochlear basilar membrane containing the organ of Corti was carefully dissected out. The specimens were rinsed in PBS three times and incubated with rabbit anti-myosin-VIIa antibody (Proteus Biosciences, USA) diluted in 1% Triton X-100 and 5% donkey serum in 0.1 M PBS (1:200) to label the body of the cochlear hair cells. After rinsing in PBS, the specimens were immersed in a secondary donkey anti-rabbit antibody conjugated with Alexa Fluor 488 diluted in PBS (1:200) for 2 h at room temperature. Then, the specimens were incubated with Alexa Fluor 568-conjugated phalloidin (Sigma, USA) for 1 h at room temperature to label the hair cell stereocilia and cuticular plate. Afterwards, each cochlear turn was mounted on glass slides in glycerin, coverslipped and examined under a fluorescent microscope (Carl Zeiss, Germany). The number of hair cells was counted along the entire length of the basilar membrane from the apex to base. Cytocochleograms were constructed by plotting the percent of outer and inner hair cell loss as a function of the percent distance from the apex of the cochlea.

### Statistical analysis

The data are presented as the means ± SEM. Repeated-measures ANOVA was performed to evaluate the difference in ABR threshold shifts, one-way ANOVA or t-test was selected to test the difference in Cox-2 expression and cochlear hair cell loss, and a Newman-Keuls post hoc test was used to assess differences between groups. Differences with a P value < 0.05 were considered statistically significant.

## Additional Information

**How to cite this article**: Sun, Y. *et al.* Inhibition of cyclooxygenase-2 by NS398 attenuates noise-induced hearing loss in mice. *Sci. Rep.* **6**, 22573; doi: 10.1038/srep22573 (2016).
